# Altered Sensory Processing in Restless Legs Syndrome: Associations With Disease Severity

**DOI:** 10.1002/brb3.71587

**Published:** 2026-07-09

**Authors:** Özgül Ocak, Erkan Melih Şahin

**Affiliations:** ^1^ Department of Neurology, Faculty of Medicine Çanakkale Onsekiz Mart University Çanakkale Türkiye; ^2^ Department of Family Medicine, Faculty of Medicine Çanakkale Onsekiz Mart University Çanakkale Türkiye

**Keywords:** altered sensory processing, disease severity, restless legs syndrome, sensory profile

## Abstract

**Objective:**

Restless legs syndrome (RLS) is increasingly recognized as a complex sensory‐motor disorder. While its motor symptoms are well‐characterized, the broader sensory processing patterns and their clinical implications remain under‐explored. This study aimed to evaluate altered sensory processing in RLS patients and investigate its relationship with disease severity and duration.

**Methods:**

This cross‐sectional study included 81 RLS patients diagnosed according to IRLSSG criteria. Diagnostic accuracy was verified through clinical records, laboratory data, and prior electromyography reports. Sensory processing was assessed using the Adolescent/Adult Sensory Profile (AASP), and RLS severity was measured with the International Restless Legs Syndrome Rating Scale (IRLS).

**Results:**

RLS patients exhibited a distinct sensory processing profile characterized by significantly higher scores in sensory sensitivity, sensory avoidance, and low registration, and lower scores in sensory seeking compared to normative data. Sensory avoidance was significantly associated with both disease severity (*r* = 0.22) and disease duration (*r* = 0.23). Females showed higher low registration scores.

**Conclusion:**

The findings suggest that altered sensory processing is a fundamental aspect of the RLS clinical phenotype. The correlation between sensory avoidance and disease severity indicates that a sensory‐based perspective may provide valuable clinical insights. Recognizing RLS as a multifaceted sensory‐motor syndrome may guide clinicians toward more comprehensive evaluation and personalized management strategies.

## Graphical abstract

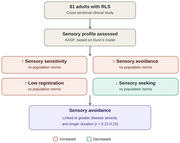

## 1 Introduction

Restless legs syndrome (RLS) is a chronic neurological disorder characterized by unpleasant sensory experiences in the legs accompanied by an urge to move, typically emerging during periods of rest and worsening in the evening or at night (Silber et al. 2021). In more than 80% of cases, somatosensory symptoms occur spontaneously and are distinct from the inner compulsion to move observed in conditions such as tics and akathisia (Antelmi et al. 2022). Psychophysical studies have demonstrated that RLS is associated with abnormalities in sensorimotor integration, including reduced pain thresholds and altered sensory processing, despite the absence of small fiber neuropathy (Bachmann et al. 2010). Emerging evidence also highlights a significant interplay between these sensory abnormalities and emotional regulation difficulties, which are increasingly recognized as part of the RLS etiology and correlate with disease severity (Kocakaya and Say 2023). Furthermore, patients with RLS may exhibit heightened vulnerability to specific psychological conditions such as alexithymia and increased suicidal ideation. It has been reported that alexithymic characteristics, particularly difficulties in identifying feelings, may be associated with higher disorder severity and serve as a contributing factor to psychiatric distress in this population (De Berardis et al. 2020). These symptoms frequently lead to sleep disturbances, impaired daytime functioning, and reduced quality of life (Engel‐Yeger et al. 2017; Engel‐Yeger 2021; Didato et al. 2020). Furthermore, external stressors and environmental triggers have been shown to exacerbate this clinical burden. For instance, the COVID‐19 pandemic significantly increased psychosocial distress and social disconnection, leading to enhanced vulnerability and reduced individual well‐being in patients with chronic conditions (Amerio et al. 2022). While it is well‐established that the prevalence of RLS increases with age and is consistently higher in women than in men (Innes et al. 2013), the underlying sensory processing mechanisms that contribute to these clinical variations—and how they relate to disease severity—remain poorly understood.

Although the etiology of RLS has not been fully elucidated, it is generally considered to involve a multifactorial interaction between genetic predisposition, dopaminergic dysfunction, and alterations in iron metabolism (Dauvilliers and Winkelmann 2013, Earley et al. 2014). In addition to these well‐established mechanisms, increasing attention has been directed toward the role of central sensory processing pathways. In particular, functional alterations involving thalamic and cortical networks have been suggested to contribute to the processing of sensory information in RLS (Kocar et al. 2020).

Dopaminergic dysfunction within central and subcortical systems plays a key role in the pathophysiology of RLS; however, its specific contribution to sensory symptoms remains incompletely understood. Structures such as the dorsolateral prefrontal cortex and basal ganglia have been implicated in this process (Yilmaz et al. 2018). Similarly, altered sensory processing may affect thalamocortical pathways responsible for transmitting and organizing sensory information. Disruptions in these pathways could potentially influence the ability to appropriately filter and respond to sensory stimuli, resulting in either hypersensitivity or reduced responsiveness (Koziol et al. 2011). Given the role of thalamocortical circuits in sensory integration, alterations in these networks may contribute to the complex sensory manifestations observed in RLS (Kocar et al. 2020).

Sensory processing refers to the ability of the nervous system to regulate and organize responses to sensory input (Brown et al. 2019). This process allows individuals to respond appropriately to environmental stimuli and plays a crucial role in daily functioning. Sensory integration involves the interpretation and integration of sensory inputs to generate appropriate motor and behavioral responses (Mulligan 2002; Brown et al. 2001). Individual variability in sensory thresholds and behavioral responses leads to differences in sensory processing patterns, and disturbances in this system may result in maladaptive responses to sensory stimuli.

The Dunn Sensory Processing Model (SPM) conceptualizes sensory processing based on the interaction between two fundamental axes: neurological thresholds and behavioral self‐regulation responses (Metz et al. 2019). Neurological thresholds represent the point at which the nervous system responds to sensory stimuli, ranging from low to high. While individuals with low thresholds are easily activated and react quickly to stimuli, those with high thresholds require more intense input to elicit a response. These thresholds can vary across different sensory modalities within the same individual. The behavioral response axis describes how individuals manage their environment in relation to these thresholds. Those with passive tendencies may react internally but do not take action to alter their surroundings, whereas individuals with active tendencies purposefully seek to control or modulate the sensory stimuli they encounter (Metz et al. 2019).

When these two axes are examined simultaneously, four distinct sensory processing patterns emerge: sensory sensitivity (low threshold, passive response), sensory avoidance (low threshold, active response), sensory seeking (high threshold, active response), and low registration (high threshold, passive response) (Dunn 2007). These patterns reflect systematic variations in how sensory information is perceived and managed, providing a framework for understanding individual differences in clinical populations.

Patients with altered sensory processing may experience challenges in interpreting bodily sensations and emotional states. In RLS, sensory symptoms are often difficult to describe, suggesting possible alterations in sensory processing mechanisms. Previous studies have reported increased sensory sensitivity in RLS patients; however, comprehensive evaluation of altered sensory processing in this population remains limited (Schattschneider et al. 2004; Antelmi et al. 2024).

Although mechanisms such as altered sensory gating or increased central responsiveness have been proposed in RLS, these concepts have not been directly demonstrated in clinical observational studies. Therefore, examining sensory processing characteristics in RLS may provide clinically relevant insights without presuming specific underlying mechanisms.

Accordingly, the primary aim of this study was to evaluate altered sensory processing in patients with RLS and to investigate the relationship between these patterns and disease severity. In line with the existing literature on sensory integration and RLS, we hypothesized that patients would demonstrate a distinct sensory processing profile compared to normative data, reflecting altered patterns across various sensory quadrants. Furthermore, we predicted that these altered sensory patterns would correlate with RLS severity and disease duration, reflecting a higher clinical burden, and that gender‐based differences in sensory processing would be observed, mirroring the established clinical prevalence of the disorder.

## 2 Methods

This study employed a cross‐sectional, correlational design. Following approval from the local ethics committee, data collection was conducted between June 15 and September 1, 2022.

The study population consisted of 81 adult patients previously diagnosed with RLS who were under regular follow‐up at the neurology outpatient clinic of the University Hospital. The diagnoses had been established by experienced neurologists in accordance with the International Restless Legs Syndrome Study Group (IRLSSG) clinical criteria during the patients' initial and follow‐up clinical evaluations. Inclusion and exclusion criteria were verified through a comprehensive review of the patients' registered medical records and previous diagnostic test results. Accordingly, the exclusion of peripheral neuropathy was based on prior electromyography (nerve conduction studies) reports available in the hospital database. Similarly, the absence of diabetes mellitus and other RLS mimics was confirmed via existing laboratory data and clinical history recorded by the treating neurology team, of which one of the researchers is a member.

To determine the sample size, a power analysis was performed using the G*Power program. Considering the primary hypothesis focused on the relationship between RLS severity and sensory processing patterns, the sample size was calculated for a bivariate correlation analysis (two‐tailed). With an *α* error level of 0.05, a power of 0.95, and a moderate‐to‐large effect size (*ρ* = 0.45) based on preliminary observations, the minimum required number of participants was determined to be 57. The final sample size of our study exceeded this requirement, providing sufficient power for both correlational and multivariate analyses.

The first section contains sociodemographic data, the second section includes scales related to sensory processing patterns, and the third section consists of questions related to the severity of RLS.

Sensory processing patterns were assessed using the Adolescent/Adult Sensory Profile (AASP), which is grounded in Dunn's SPM (Brown and Dunn 2002). The AASP consists of 60 items rated on a 5‐point Likert scale ranging from 1 (*almost never*) to 5 (*almost always*). Each of the four quadrants—sensory sensitivity, sensory avoidance, sensory seeking, and low registration—results in a score ranging from 15 to 75. Participants are classified based on predetermined norm values as “much less than most people,” “less than most people,” “similar to most people,” “more than most people,” and “much more than most people.” Deviations from the norm in any quadrant, whether in the direction of higher or lower scores, are interpreted as distinct sensory processing characteristics (Dunn 2007; Brown and Dunn 2002).

The clinical severity of RLS was evaluated using the IRLS, which is a globally recognized and standardized clinical tool (Hening and Allen 2003; Walters et al. 2003). The scale was developed based on criteria recommended by members of the IRLSSG with extensive clinical expertise (Walters et al. 2003). This instrument consists of 10 items assessing the subjective evaluation of main characteristics (Questions 1, 2, and 3), the severity and frequency of the disease (Questions 7 and 8), and related sleep problems (Questions 4 and 5). The scale also includes questions that investigate the impact of symptoms on patients' mental status and daily functions (Questions 9 and 10). Each question in the IRLS is assigned a value representing the severity of RLS symptoms, ranging from no impact (0 points) to very severe RLS symptoms (4 points). Thus, the total score obtained from the scale ranges from 0 to 40. Scores in the range of 1–10 indicate mild RLS, scores in the range of 11–20 indicate moderate RLS, scores in the range of 21–30 indicate severe RLS, and scores in the range of 31–40 indicate very severe RLS (Hening and Allen 2003; Walters et al. 2003).

### 2.1 Statistical Analysis

Descriptive statistics were reported as frequencies, percentages, means, and standard deviations. Normality was confirmed through visual inspection (histograms and *Q–Q* plots) and skewness/kurtosis values. Based on these assessments and the Central Limit Theorem, parametric tests were employed (Rice 2006). The primary hypotheses of the study, focusing on the relationship between RLS severity and sensory processing patterns, were tested using Pearson correlation coefficients. The strength of the correlations was interpreted based on Cohen's criteria, where absolute values of *r* = 0.10–0.29 were considered a small effect, *r* = 0.30–0.49 a medium effect, and *r* ≥ 0.50 a large effect (Cohen 1988). To further investigate demographic variations such as sex‐related differences across the four interdependent quadrants of the AASP, multivariate analysis of variance (MANOVA) was conducted. Test statistics and absolute *p*‐values were provided, and a general significance level of *p* < 0.05 was accepted for all analyses; however, a Bonferroni correction was applied to account for multiple comparisons, and the significance threshold was adjusted to *p* < 0.0125.

This manuscript has been translated into English using the OpenAI language model, GPT‐4. The model provided linguistic assistance in translating the original text and ensuring the accuracy and clarity of the English version. The authors have reviewed the translation for content accuracy and have made necessary adjustments to ensure the scientific integrity of the manuscript.

## 3 Results

The demographic characteristics of the participants, including age, sex, educational status, and occupational status, are summarized in Table 1. There was no significant difference in age between females (52.07 ± 12.46 years) and males (51.54 ± 15.31 years) (*t* = 0.163; *p* = 0.871). The educational status of males was significantly better than that of females (*U* = 415.0; *p* = 0.004).

**TABLE 1:** Participants’ sociodemographic information.

**Gender**	
Female	57 (70.4%)
Male	24 (29.6%)
**Age**	51.91 ± 13.27 (range: 20–78)
**Educational status**	
Primary school	23 (28.4%)
Secondary school	7 (8.6%)
High school	23 (28.4%)
Higher education	28 (34.5%)
**Occupational status**	
Active worker	21 (25.9%)
Not working actively	28 (34.6%)
Retired	30 (37.0%)
Unemployed	2 (2.5%)

The participants had been diagnosed with RLS for an average of 4.94 ± 4.54 years (min: 1, max: 18). There was no significant difference in the duration of diagnosis between females (5.15 ± 4.60 years) and males (4.43 ± 4.46 years) (*t* = 0.629; *p* = 0.532). There was no significant correlation between the participants' ages and the duration of diagnosis (*r* = 0.182; *p* = 0.112).

The mean score on the IRLS for participants was 23.85 ± 7.59 (ranging from 2 to 38). Based on these scores, disease severity was classified as mild in four (4.9%) participants, moderate in 20 (24.7%) participants, severe in 40 (49.4%) participants, and very severe in 17 (21.0%) participants.

There was no significant difference in the mean IRLS scores between genders (*t* = 0.879; *p* = 0.382). There was no significant correlation between the participants' ages and their IRLS scores (*r* = 0.064; *p* = 0.571). A significant negative correlation was found between the participants' educational status and the severity groups of RLS (*r* = −0.231; *p* = 0.038).

The mean score on the AASP was 154.47 ± 27.03 (ranging from 71 to 202). Details regarding the axes of Dunn's SPM are presented in Table 2.

**TABLE 2:** Scores and distribution of participants into categories regarding the axes of Dunn's Sensory Processing Model.

	Sensory sensitivity	Sensory avoidance	Low registration	Sensory seeking
Scale score (Mean ± SD)	41.67 ± 9.94	40.57 ± 9.21	34.16 ± 9.49	38.07 ± 7.14
Much less than most people	1 (1.2%)	1 (1.2%)	3 (3.7%)	31 (38.3%)
Less than most people	4 (4.9%)	7 (8.6%)	10 (12.3%)	29 (35.8%)
Similar to most people	33 (40.7%)	32 (39.5%)	32 (39.5%)	21 (25.9%)
More than most people	22 (27.2%)	28 (34.6%)	25 (30.9%)	0 (0.0%)
Much more than most people	21 (25.9%)	13 (16.0%)	11 (13.6%)	0 (0.0%)

*Note*: Data are presented as mean ± standard deviation (SD) or number (percentage). The categories are based on the Adolescent/Adult Sensory Profile (AASP) norms.

Abbreviation: SD, standard deviation.

In the *sensory sensitivity* dimension, 53.1% of the participants were classified in the “more than most people” and “much more than most people” categories, indicating a heightened sensory responsiveness compared to the general population. This finding is consistent with a low neurological threshold, where sensory stimuli are detected more easily and lead to exaggerated reactions.

In the *sensory avoiding* dimension, more than half of the participants (50.6%) showed increased avoidance behaviors in response to sensory input. This suggests that individuals with RLS may adopt behavioral strategies to minimize exposure to overwhelming sensory stimuli due to intolerance of sensory overload.

For the *low registration* dimension, approximately 44.5% of individuals demonstrated difficulties in noticing or responding to sensory stimuli. When interpreted alongside the increased sensory sensitivity, this indicates a complex sensory processing profile in which individuals may be both hypersensitive to certain stimuli yet fail to effectively register others.

Conversely, in the *sensory seeking* dimension, the majority of participants (74.1%) were categorized as “less than most people” or “much less than most people,” and no participants were classified in the “more than most” or “much more than most” categories for this dimension. This outcome highlights a marked reduction in active engagement with sensory experiences and supports the dominance of avoidance‐oriented behavioral responses.

A one‐way MANOVA was conducted to examine gender differences across the four sensory processing patterns. Results indicated that the multivariate effect of gender on the combined dependent variables did not reach statistical significance (Wilks’ *Λ* = 0.890, *F* = 2.344, *p* = 0.062). Follow‐up univariate analyses, using a Bonferroni‐corrected *α* level of 0.0125, revealed that only the difference in low registration reached statistical significance (*F* = 9.226, *p* = 0.003), with women scoring higher than men. Although women also had higher scores in sensory sensitivity (*F* = 5.992, *p* = 0.017) and sensory avoidance (*F* = 4.881, *p* = 0.030), these differences did not meet the stringent Bonferroni‐adjusted threshold. No significant difference was observed in the sensory seeking axis (*F* = 0.031, *p* = 0.860). There was no significant correlation between the participants' ages and their scores on the SPM axes.

A significant weak positive correlation was found between disease duration and the sensory avoidance axis scores of the SPM (*r* = 0.228; *p* = 0.046). There were no significant correlations between sensory sensitivity, low registration, and sensory seeking axis scores and disease duration (*r* = 0.196; *p* = 0.088, *r* = 0.132; *p* = 0.251, *r* = 0.091; *p* = 0.430, respectively).

Similarly, a significant weak positive correlation was found between IRLS scores and the sensory avoidance axis scores of the SPM (*r* = 0.223; *p* = 0.045). There were no significant correlations between IRLS scores and sensory sensitivity, low registration, and sensory seeking axis scores of the SPM (*r* = 0.201; *p* = 0.072, *r* = 0.164; *p* = 0.143, *r* = 0.147; *p* = 0.189, respectively).

**FIGURE 1:**
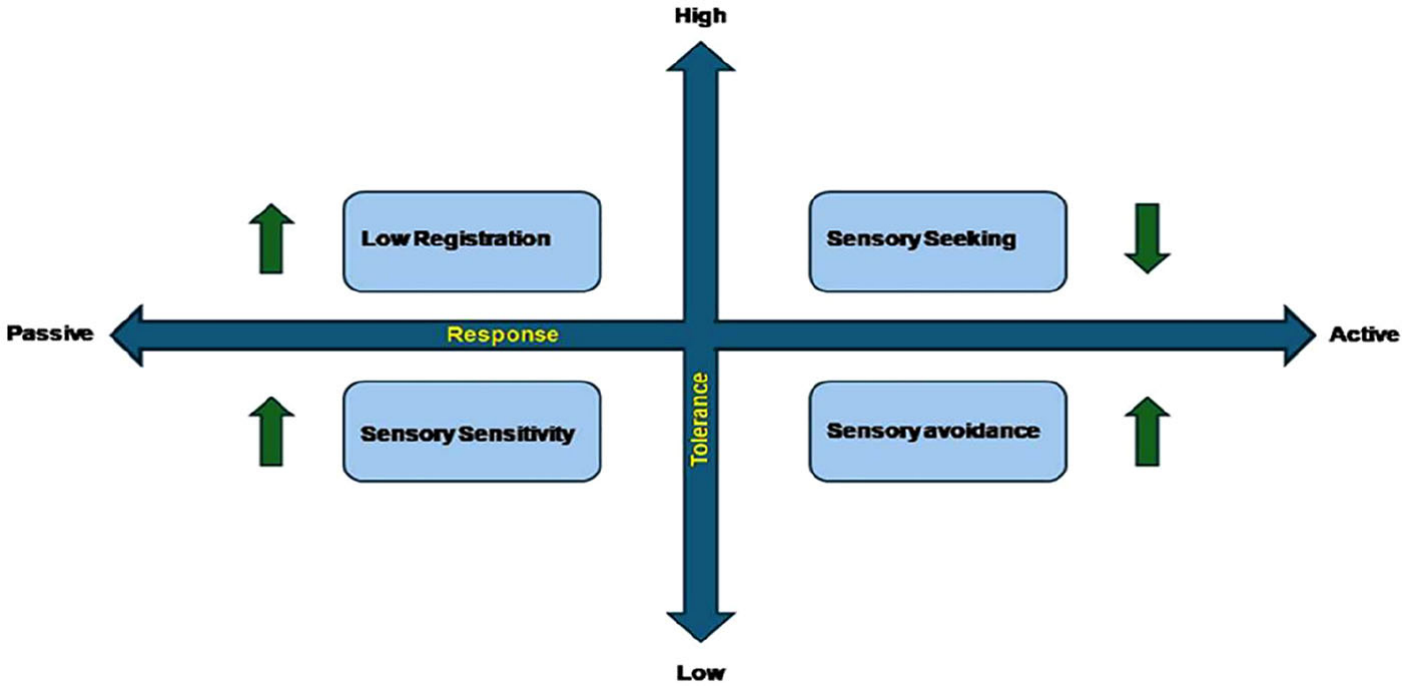
Altered sensory processing profile in patients with restless legs syndrome (RLS) based on Dunn's Sensory Processing Model.

The AASP, based on Dunn's SPM, was used to assess sensory processing profiles. This model conceptualizes sensory processing through the interaction of two primary continua: neurological threshold (the intensity of stimulation required for neuronal activation) and behavioral response (passive–active coping strategies). The intersection of these dimensions defines four core sensory processing patterns: low registration, sensation seeking, sensory sensitivity, and sensory avoidance. This conceptual framework and the schematic representation of these patterns are illustrated in Figure 1.

The diagram illustrates the four quadrants of sensory processing, defined by the interaction between neurological threshold (vertical axis) and behavioral response (horizontal axis). Upward arrows (↑) indicate that patients with RLS scored higher than population norms in sensory sensitivity, sensory avoidance, and low registration, while the downward arrow (↓) indicates reduced sensory seeking. This pattern reflects a profile characterized by increased sensitivity and avoidance behaviors accompanied by reduced engagement in active stimulus‐seeking.

## 4 Discussion

This study provides a comprehensive evaluation of altered sensory processing in patients with RLS and demonstrates that individuals with RLS exhibit a distinct sensory processing profile characterized by increased sensory sensitivity, sensory avoidance, and low registration, along with reduced sensory seeking behaviors. These findings provide preliminary evidence suggesting that alterations in sensory processing may contribute to the multifaceted clinical presentation of RLS.

Sensory processing plays a fundamental role in regulating responses to environmental and internal stimuli (Gardner 2016). Disruptions in this process may lead to maladaptive behavioral patterns, including hypersensitivity or reduced responsiveness to sensory input (Miller et al. 2007). In our cohort, patients with RLS demonstrated higher sensory sensitivity, sensory avoidance, and low registration scores compared to normative values.

According to Dunn's model, individuals with a low neurological threshold tend to detect sensory stimuli more readily and respond to them more intensely (Costa‐López et al. 2021).

Sensory symptoms reported by patients with RLS often do not conform to conventional sensory categories and are typically described as vague, uncomfortable, or difficult‐to‐define internal sensations. This observation supports previous findings suggesting that sensory experiences in RLS represent a heterogeneous and atypical spectrum rather than clearly defined nociceptive or somatic modalities (Winkelman et al. 2013).

Previous studies have suggested the presence of neurophysiological alterations in sensory processing in RLS, including changes in cortical excitability and the processing of sensory inputs (Yang et al. 2019). Similarly, some models propose that functional alterations in central sensory processing networks may contribute to the clinical features of RLS (Ferré et al. 2019).

A notable finding of this study was the coexistence of increased sensory sensitivity and low registration. This pattern suggests that patients may be highly responsive to certain stimuli while simultaneously showing reduced awareness of others.

Gender‐related differences were also observed, with female patients demonstrating higher low registration scores compared to males. These findings are consistent with previous literature indicating sex‐related differences in sensory processing (Schaaf and Lane 2015). In addition, RLS has been reported to be more prevalent in women, and some studies suggest that symptom severity may be greater in female patients, potentially related to hormonal and biological factors (Schaaf and Lane 2015). In this context, the altered sensory processing observed in female patients in our study—particularly the significantly higher low registration scores—suggests that sex‐related differences in RLS may not be solely explained by biological mechanisms but may also be associated with distinct sensory processing profiles. Similar sensory processing profiles—often characterized by increased sensitivity, avoidance, or registration deficits—have been described in various clinical conditions involving altered sensory processing. Therefore, our findings provide a novel perspective by indicating that sensory processing characteristics may contribute to the observed sex differences in RLS and should be considered in future evaluations.

Additionally, sensory avoidance was positively associated with both disease severity and disease duration. While the observed correlation coefficients (*r* = 0.22) indicate a small effect size, this finding is highly significant considering the multifactorial etiology of RLS. The persistence of this association across both duration and severity markers suggests that sensory processing characteristics are inherently related to the accumulative clinical burden of the disorder.

While similar associations between persistent sensory input and increased central responsiveness have been described in other clinical conditions, the demonstration of this relationship in the context of RLS is presented for the first time in this study (Baron et al. 2013). From a clinical perspective, these findings suggest that altered sensory processing may play a role in the symptom expression of RLS and suggest the potential utility of considering sensory‐based perspectives in clinical evaluation. Overall, these preliminary results suggest that RLS involves not only motor symptoms but also broader alterations in sensory processing, supporting a more integrated understanding of the disorder's sensory‐motor phenotype.

Furthermore, the co‐existence of patterns such as sensory sensitivity and low registration in RLS patients suggests a complex self‐regulatory dynamic. As highlighted by recent perspectives, the motor restlessness and associated sensory stimulation in RLS may serve as a compensatory strategy to achieve emotional and sensory relaxation. In this context, for individuals with high neurological thresholds (low registration), the intense proprioceptive and tactile input generated by movement may provide the necessary sensory “load” to reach a state of homeostatic balance (Dunn 1997). Conversely, for those with low thresholds (hypersensitivity), these self‐generated movements might act as a rhythmic masking mechanism to modulate or “drown out” distressing internal or external stimuli, thereby potentially serving as a subconscious strategy for anxiety reduction and emotional regulation (Hening et al. 2004). This view positions RLS symptoms not merely as manifestations of neurological dysfunction but also as an active, albeit disruptive, attempt by the nervous system to achieve sensory and emotional regulation.

A key strength of our study is that, to our knowledge, it is the first to systematically evaluate altered sensory processing in patients with RLS and to empirically demonstrate their relationship with disease severity, reinforcing our primary hypothesis that altered sensory processing is a fundamental aspect of the condition.

Despite the significant findings, our study has several limitations that should be considered. First, the cross‐sectional design of the research precludes drawing definitive causal inferences regarding the relationship between RLS and sensory processing alterations. Second, although the sample size provided sufficient power to detect the reported effects, the single‐center nature of the study may limit the generalizability of the findings to more diverse clinical settings and different cultural populations. Third, both sensory processing and disease severity were assessed using self‐report measures (AASP and IRLS), which may introduce subjective bias or social desirability bias into the data. Finally, the potential influence of pharmacological treatments (e.g., dopamine agonists or alpha‐2‐delta ligands) on sensory processing patterns was not controlled for in the current analysis.

## 5 Conclusion

In conclusion, our study demonstrates that RLS is associated with a distinct sensory processing profile characterized by heightened sensory sensitivity, sensory avoidance, and low registration. The observed relationship between sensory avoidance and disease severity suggests that altered sensory processing may represent an important aspect of the RLS clinical phenotype. The primary clinical message of this research is that expanding the evaluation of RLS beyond motor symptoms to include sensory processing patterns may provide a more comprehensive understanding of the patient's experience. Specifically, the recognition of sensory avoidance patterns may guide clinicians in identifying patients who may experience a higher clinical burden. These preliminary findings provide a basis for future research to explore the potential role of sensory‐based perspectives as adjuncts to traditional management. Ultimately, viewing RLS through a sensory‐motor lens, rather than as a movement disorder alone, may contribute to more holistic approaches in clinical evaluation.

## Author Contributions

Conception and design: Özgül Ocak and Erkan Melih Şahin. Data collection: Özgül Ocak and Erkan Melih Şahin. Interpretation of the data and analysis: Özgül Ocak and Erkan Melih Şahin. Drafting: Özgül Ocak and Erkan Melih Şahin. Revising: Özgül Ocak and Erkan Melih Şahin. The manuscript has been read and approved by all the authors.

## Funding

The authors have nothing to report.

## Disclosure

This manuscript is based on original work and had not been published in whole or part, in any print or electronic media or is under consideration of publication in any print or electronic media other than as abstract of conference proceedings. This manuscript had not been presented in whole or part, in any scientific meeting orally or as a poster. This manuscript does not contain any reproduced material from other sources.

## Ethics Statement

Ethics committee approval was obtained for the study from Çanakkale Onsekiz Mart University Ethics Committee for Clinical Studies with decision number 2022‐10/18 dated June 1, 2022. The study was conducted in accordance with the latest version of the Declaration of Helsinki.

## Consent

Verbal informed consent was obtained from all patients included in this study prior to the interview.

## Conflicts of Interest

The authors declare no conflicts of interest.

## Data Availability

The datasets generated during and/or analyzed during the current study are available from the corresponding author on reasonable request.
